# Circulating tumor cells as a prognostic factor in patients with small cell lung cancer

**DOI:** 10.3892/ol.2014.1940

**Published:** 2014-03-05

**Authors:** SATOSHI IGAWA, KEIGO GOHDA, TOMOYA FUKUI, SHINICHIRO RYUGE, SAKIKO OTANI, AKINORI MASAGO, JUN SATO, KATSUHIRO MURAKAMI, SACHIYO MAKI, KEN KATONO, AKIRA TAKAKURA, JIICHIRO SASAKI, YUKITOSHI SATOH, NORIYUKI MASUDA

**Affiliations:** 1 Department of Respiratory Medicine, Kitasato University School of Medicine, Sagamihara, Kanagawa 252-0374, Japan; 2 Central Research Laboratories, Sysmex Corporation, Kobe, Hyōgo 651-2271, Japan; 3 Department of Thoracic Surgery, Kitasato University School of Medicine, Sagamihara, Kanagawa 252-0374, Japan

**Keywords:** circulating tumor cells, small cell lung cancer, prognostic factor, OBP-401 assay

## Abstract

The detection of circulating tumor cells (CTCs) in peripheral blood is currently an important field of study. Detection of CTCs by the OBP-401 assay (TelomeScan^®^) has previously been reported to be useful in the diagnosis, prognosis and evaluation of therapeutic efficacy in breast and gastric cancer. The aim of the present study was to evaluate the OBP-401 assay as a novel method of detecting CTCs of small cell lung cancer (SCLC) patients and to evaluate whether CTC count is associated with prognosis. Prospectively, 30 consecutively diagnosed SCLC patients who had commenced chemotherapy or chemoradiotherapy were enrolled as subjects of the current study. Peripheral blood specimens were collected from the SCLC patients prior to and following the initiation of treatment and the viable CTCs were detected in the specimens following incubation with a telomerase-specific, replication-selective, oncolytic adenoviral agent, which was carrying the green fluorescent protein gene. CTCs were detected in 29 patients (96%). The group of 21 patients with a CTC count of <2 cells/7.5 ml prior to treatment (baseline) had a significantly longer median survival time than the group of eight patients with a CTC count of ≥2 cells/7.5 ml prior to treatment (14.8 and 3.9 months, respectively; P=0.007). The results of a multivariate analysis showed that the baseline CTC count was an independent prognostic factor for survival time (hazard ratio, 3.91; P=0.026). Among the patients that achieved a partial response to treatment, patients who had a CTC count of <2 cells/7.5 ml following two cycles of chemotherapy tended to have a longer median progression-free survival compared with patients who had a CTC count of ≥2 cell/7.5 ml (8.3 and 3.8 months, respectively; P=0.07). Therefore, CTCs may be detected via OBP-401 assay in SCLC patients and the CTC count prior to treatment appears to be a strong prognostic factor.

## Introduction

Primary lung cancer is the leading cause of cancer-related mortality in the majority of industrialized countries and ~15% of primary lung cancer patients have small cell lung cancer (SCLC) (1,2). In total, 60–70% of SCLC patients present with extensive-stage disease (2). Following apparently successful induction therapy, the majority of patients are diagnosed as experiencing a relapse within two years due to the emergence of drug-resistant cancer cells, during the induction therapy, or due to their presence prior to chemotherapy. As a result, long-term survival is quite uncommon; <25% of patients with limited-stage disease and only 1–2% of patients with extensive-stage disease survive for five years (3,4). The high mortality rate of SCLC is predominantly attributable to the frequent recurrence of distant metastasis. Thus, tumor cells may be circulating in the blood of the majority of SCLC patients at the time of diagnosis irrespective of whether there is any clinical evidence of distant metastasis or not. The CellSearch System^®^ (Veridex LLC, Raritan, NJ, USA) is a commercially available system for detecting circulating tumor cells (CTCs). Previous studies have shown that CTCs may be detected in the peripheral blood of approximately half of lung cancer patients using the CellSearch System^®^. Detection of CTC presence and their characteristics may be used to estimate the risk of metastatic relapse, facilitate stratification of patients to adjuvant therapy, select therapeutic regimens and monitor the efficacy of systemic anticancer therapy (5–7). Taki *et al* (8) previously constructed an adenovirus vector termed Telomelysin^®^ (OBP-301^®^) that drives the *E1A* and *E1B* genes under the control of the human telomerase reverse transcriptase (hTERT) promoter, and demonstrated its selective and supersensitive replication in a variety of viable human cancer cells. The authors developed a telomerase-specific replication-selective adenovirus OBP-401 assay (TelomeScan^®^; Oncolys BioPharma Inc., Tokyo, Japan), in which the green fluorescent protein (GFP) gene is driven by a cytomegalovirus promoter (9). Telomerase is a ribonucleoprotein complex that is responsible for the complete replication of chromosomal ends (10,11). Expression of telomerase activity has been demonstrated in >85% of human cancers, however, only in limited numbers of normal somatic cells (12,13). Therefore, telomerase activation is considered to be a critical step in carcinogenesis and telomerase activity has been found to closely correlate with hTERT expression (14). Subsequently, the present study was conducted to assay the peripheral venous blood of SCLC patients for the presence of CTCs, using the novel OBP-401 assay, and investigate whether the CTC count of peripheral venous blood was associated with prognosis.

## Patients and methods

### Study design

The current prospective study was conducted at the Kitasato University Hospital (Sagamihara, Japan). The recruitment criteria were as follows: i) Histologically or cytologically confirmed SCLC and confirmation of the clinical stage based on the results of examination by chest X-ray, computed tomography (CT) of the chest and abdomen. In addition to other procedures as indicated, including brain magnetic resonance imaging (MRI) and positron emission tomography (PET) scanning or radionuclide bone scanning; ii) chemotherapy-naïve; iii) evaluable or measurable disease; iv) adequate bone marrow, hepatic and renal function; v) no active concomitant malignancy; and vi) written informed consent. Tumor response was classified in accordance with the Response Evaluation Criteria for Solid Tumors. CT, brain MRI and PET scanning or radionuclide bone scanning were routinely conducted to evaluate tumor progression. The institutional review board at the Kitasato University Hospital approved the study protocol and all patients provided written informed consent.

A peripheral blood specimen was collected to analyze the presence of CTCs at each of the following five time periods: Within seven days prior to commencing treatment (baseline); chemotherapy cycles two and three; following chemotherapy cycle four; and the time point at which progressive disease was confirmed.

### CTC detection

The OBP-401 virus was used to detect CTCs as described previously (15). Briefly, a 7.5-ml peripheral-vein blood sample was drawn into tubes containing citric acid, phosphoric acid and dextrose (Nacalai Tesque, Inc., Kyoto, Japan). The himac CF12RX (Hitachi, Ltd., Tokyo, Japan) was used for centrifugation at 540 × g for 5 min. Following washing with phosphate-buffered saline (PBS) and RPMI-1640 medium (Sigma-Aldrich, St. Louis, MO, USA) by centrifugation, the samples were infected with a 4×10^8^ plaque-forming unit of OBP-401 virus for 24 h at 37°C. Each sample was fixed with 4% paraformaldehyde (Wako Pure Chemical Industries, Osaka, Japan) and treated with a surface-active agent (Emalgen 2025 G; Kao Chemicals, Tokyo, Japan) to degrade the red blood cells. The remaining cells were scraped from the slides, applied to glass slides and examined using a fluorescence microscope (IX71; Olympus Corporation, Tokyo, Japan).

### Immunostaining

The glass slides were soaked in PBS to remove the coverglass and the cells were scraped from the slides, suspended in PBS and applied to glass slides by cytocentrifugation in a Cytospin 4 cytocentrifuge (Thermo Fisher Scientific, Waltham, MA, USA). The cells were subsequently blocked with blocking buffer [1% normal goat serum (Millipore, Bedford, MA, USA) in PBS] for 1 h at room temperature and incubated for 1 h at room temperature with mouse monoclonal anti-pan cytokeratin antibody (ab961; Abcam, Cambridge, UK). Next, the cells were washed with PBS and treated with Alexa Fluor 405-conjugated secondary antibody (Invitrogen Life Technologies, Carlsbad, CA, USA) for 1 h at room temperature. Following washing with PBS, the cells were mounted and examined using a fluorescence microscope (IX71; Olympus, Tokyo, Japan) and the number of CTCs in the 7.5 ml peripheral venous blood was counted.

### Definition of CTC

**Figure 1:**
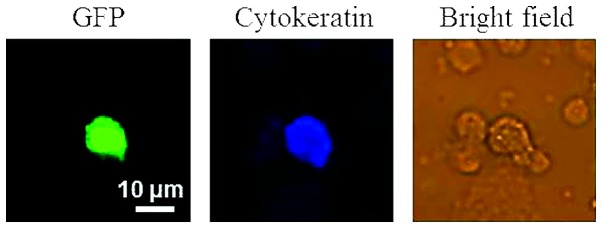
Immunocytochemical analysis of a GFP-positive cell from a small cell lung cancer patient. The glass slides of circulating tumor cells were processed by immuocytochemical analysis. Cytokeratin was detected with anti-pan cytokeratin antibody (magnification, ×20). GFP, green fluorescent protein.

Previously, GFP-positive cells with relatively small diameters have been observed in blood samples obtained from cancer patients and healthy controls (16). The small GFP-positive cells were double-stained with a variety of anti-CD antibodies (CD2/3/13/14/15/16/19/45/203c; Biolegend, Inc., San Diego, CA, USA) to characterize the cell attributes and the results showed that the majority (~70%) of the small GFP-positive cells were monocytes (16). Accordingly, to define CTCs in the blood samples of cancer patients, the cut-off value of cell diameter for CTCs was determined as >8.4 μm. This value was deduced from the average value plus two standard deviations, by analyzing the diameter distribution of monocytes in the blood samples of healthy controls, which statistically indicated that >95% of monocytes may be excluded from the GFP-positive cells in the blood samples of cancer patients. Consequently, in the present study, GFP-positive cells >8.4 μm in diameter were counted as CTCs (17). A GFP-positive cell that was isolated from the peripheral blood of a SCLC patient is shown in Fig. 1.

### Statistical analysis

The primary analysis was a comparison between overall survival (OS) in the unfavorable and favorable groups stratified according to the selected threshold of baseline CTC count. OS and progression-free survival (PFS) were measured from the date of when the baseline blood sample was collected to the date when clinical progression was confirmed by mortality or censoring at the last follow-up examination. PFS and OS curves were plotted using the Kaplan-Meier method and differences in survival time were analyzed for statistical significance with the log-rank test. Student’s t-test was performed to evaluate absolute change in the mean CTC count. Cox proportional hazards regression was used to determine the hazard ratios (HRs) for OS, which were adjusted for age, gender, pretreatment stage (extensive disease versus limited disease), Eastern Cooperative Oncology Group performance status, serum lactate dehydrogenase (LDH) levels, serum Na levels and the baseline CTC count prior to the initiation of chemotherapy. P<0.05 was considered to indicate a statistically significant difference and the statistical analysis was performed using the SPSS for Windows software program, version 17 (SPSS, Inc., Chicago, IL, USA).

## Results

### Patient characteristics

The 30 patients with histologically or cytologically confirmed SCLC (28 males and two females; mean age, 69 years; age range, 51–85 years; limited disease patients, n=8; and extensive disease patients, n=22) who met the inclusion criteria between April 2009 and December 2011 were the subjects of the current study. The patient characteristics are summarized in Table I. At the time of analysis, 28 of the 30 (93%) evaluable patients had experienced disease progression, and 25 of the 30 (83%) evaluable patients had succumbed to their diseases, resulting in a median PFS of 5.7 months (95% CI, 4.8–6.5 months) and median survival time of 11.5 months (95% CI, 9.2–13.7 months). The median follow-up period for determining the survival time was 12.0 months from the baseline blood sample collection.

### Evaluating the change in CTC counts between baseline and after two cycles of chemotherapy

CTCs were detected in 29 of the 30 (96%) patients. The blood samples following two cycles of chemotherapy (prior to chemotherapy cycle three) for CTC analysis were obtained from 29 patients. The comparison between the mean CTC count at baseline and following two cycles of chemotherapy, according to treatment response, are shown in Table II. Among the patients who exhibited a partial response (PR) following two cycles of chemotherapy, the mean CTC count tended to increase (2.32 cells/7.5 ml) compared with the mean CTC count at baseline (0.84 cells/7.5 ml) regardless of a reduction in tumor volume (P=0.05).

### Correlation between the GFP-positive CTC count and survival time

**Figure 2:**
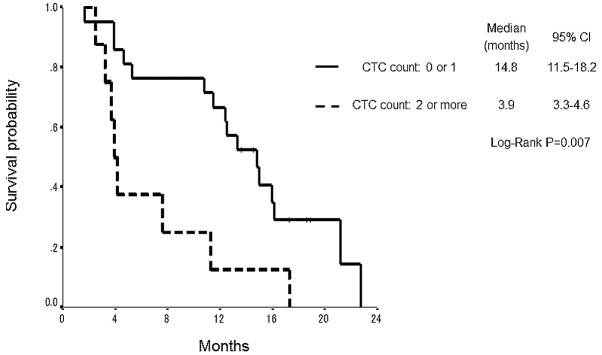
Overall survival of patients according to the CTC count at baseline. CTC, circulating tumor cell; CI, confidence interval.

**Figure 3:**
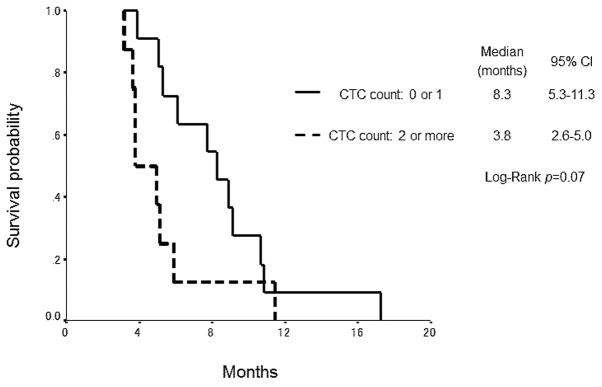
Progression-free survival according to the CTC count of patients with a partial response following two cycles of chemotherapy. CTC, circulating tumor cell; CI, confidence interval.

The group of 21 patients with CTC counts of <2 cells/7.5 ml at the baseline exhibited a significantly longer median survival time (14.8 months; 95% CI, 11.5–18.2) than the group of nine patients with a CTC count of ≥2 cells/7.5 ml (3.9 months; 95% CI, 3.3–4.6) (P=0.007; Fig. 2). The group of patients that exhibited a PR following two cycles of chemotherapy who had a CTC count of <2 cells/7.5 ml prior to chemotherapy cycle three tended to have a longer median PFS (8.3 months; 95% CI, 5.3–11.3) compared with the group with a CTC count of ≥2 cells/7.5 ml (3.8 months; 95% CI, 2.6–5.0) (P=0.07; Fig. 3).

### Univariate survival analyses and multivariate Cox proportional hazards regression analysis

The clinical factors that were identified as significant in relation to survival time in the univariate analysis were stage at diagnosis, serum LDH levels and baseline CTC count (Table III). Multivariate analysis with adjustment for these factors identified all of the factors (HR, 3.91; 95% CI, 1.19–12.87; P=0.026) to be independent prognostic markers for OS (Table IV).

## Discussion

Detection of CTCs by the OBP-401 assay has previously been reported to be useful in the diagnosis, prognosis and evaluation of therapeutic efficacy in breast and gastric cancer (15,16,18). With regard to SCLC, previous studies have reported that higher CTC counts detected by the CellSearch^®^ System were strongly associated with shorter SCLC patient survival times (19–21). The present study was, to the best of our knowledge, the first to report the detection of CTCs in patients with SCLC using the OBP-401 assay, as well as being the first prospective evaluation of CTCs used to predict a prognosis in SCLC patients with the OBP-401 assay.

Various SCLC patients, such as responders or non-responders to chemotherapy, were inevitably included prior to commencing treatment. Therefore, attention was restrictively focused on the CTC counts of patients who had acquired PR following two cycles of chemotherapy. The patients who responded to chemotherapy included one group in which the CTC count increased and another group in which the CTC count decreased compared with the baseline group of responders, regardless of the reduction in tumor volume. Additionally, the group of responders with a CTC count of ≥2 tended to show a shorter PFS compared with the group of responders with a CTC count of <2 cells/7.5 ml. Accordingly, it is reasonable to suppose that the CTC count following two cycles of chemotherapy may be a predictor for the duration of relapse-free time among responders.

Immunomagnetic cell enrichment, such as that performed by the CellSearch^®^ system, is currently the most commonly used technique to detect CTCs (5–7). In this assay, cells detected with antibodies against epithelial markers (epithelial cell adhesion molecule [EpCAM]) are determined to be CTCs. Previous studies have reported that epithelial mesenchymal transition (EMT) is important in the process of vascular invasion by tumor cells and results in hematogenous dissemination (22,23). Thus, it is considered that a significant number of CTCs are EMT tumor cells and that the percentage of CTCs that are EMT tumor cells may vary from patient to patient. Since EMT tumor cells have been reported to only weakly express epithelial surface antigens, including EpCAM, EMT tumor cells are less likely to be detected by the Cell Search^®^ System. The comparison between the OBP-401 assay and CellSearch^®^ system for analyses of CTCs showed that the OBP-401 assay does not include the enrichment process of epithelial surface antigens, including EpCAM. The OBP-401 assay identifies CTCs on the basis of telomerase expression irrespective of epithelial surface antigen expression. Accordingly, the OBP-401 assay must be more suitable for the detection of EMT tumor cells (23–27).

The major limitations of the current study were that the study population was small and the study was performed at a single institution. Thus, the threshold value of the CTC count as a prognostic factor was derived on the basis of results from a study population at the Kitasato University Hospital alone and was not independently validated at an additional institution. In addition, the study population included patients treated by chemoradiotherapy as well as patients who were treated by chemotherapy alone. Since the purposes of chemotherapy and chemoradiotherapy are different, separate derivation studies are required to determine the optimal threshold value of the CTC count.

In conclusion, the results of the current study showed that CTCs may be detected by the OBP-401 assay in SCLC patients and that the CTC count prior to treatment may be a strong prognostic factor. A large prospective multi-institutional validation study is required to confirm these results.

## Figures and Tables

**Table I:** Characteristics of 30 patients with histologically or cytologically confirmed small cell lung cancer.

Patient characteristics	Value
Age at baseline, years	
Median	69
Range	51–85
Gender, n (%)	
Male	28 (93)
Female	2 (7)
Stage at diagnosis, n (%)	
Limited disease	8 (27)
Extensive disease	22 (73)
Baseline WHO PS, n (%)	
0 or 1	20 (66)
2	8 (27)
3	2 (7)
Treatment received, n (%)	
Combination regimen	
Carboplatin + etoposide	11 (36)
Cisplatin + etoposide	2 (7)
Cisplatin + irinotecan	1 (3)
Carboplatin + irinotecan	2 (7)
Amrubicin + irinotecan	8 (27)
Single agent regimen, n (%)	
Amrubicin	6 (20)
Laboratory value, n (%)	
Na, meq/l	
≥135	9 (30)
<135	21 (70)
LDH, U/l	
≥229	10 (33)
<229	20 (67)

WHO, World Health Organization; PS, performance status; Na, sodium; LDH, lactate dehydrogenase.

**Table II:** CTC count according to treatment response following two cycles of chemotherapy.

Effect of treatment	n	CTCs base line (cells/7.5 ml)	CTCs^a^ (cells/7.5 ml)	P-value
PR	19	0.84	2.32	0.05
SD/PD	10	1.67	2.76	0.41

a CTC count following two cycles of chemotherapy.

CTC, circulating tumor cell; PR, partial response; SD, stable disease; PD, progressive disease.

**Table III:** Univariate Cox regression analysis for prediction of OS.

	OS rate		
	
Parameter	P-value	HR	95% CI
Gender	0.84	1.23	0.16–9.26
M			
F			
Age, years	0.17	2.07	0.73–5.89
<75			
≥75			
PS, n	0.16	1.95	0.77–4.99
0 or 1			
≥2			
Na, meq/l	0.15	1.98	0.79–4.92
<135			
≥135			
LDH, U/l	0.001	12.82	2.70–60.61
<229			
≥229			
Stage at diagnosis	0.014	12.52	1.68–93.46
Limited			
Extensive			
CTC count at baseline	0.028	2.96	1.13–7.76
0 or 1			
≥2			

OS, overall survival; HR, hazard ratio; CI, confidence interval; M, male; F, female; Na, sodium; LDH, lactate dehydrogenase; CTC, circulating tumor cell.

**Table IV:** Stepwise multivariate Cox regression analysis for prediction of OS.

	OS rate		
	
Parameter	P-value	HR	95% CI
LDH, U/l	0.012	0.13	0.027–0.64
<229			
≥229			
Stage at diagnosis	0.023	0.090	0.011–0.71
Extensive			
Limited			
CTC at baseline	0.026	3.91	1.19–12.87
0 or 1			
≥2			

OS, overall survival; HR, hazard ratio; CI, confidence interval; LDH, lactate dehydrogenase; CTC, circulating tumor cell.
